# Impact of mannose-binding lectin deficiency on radiocontrast-induced renal dysfunction: a post-hoc analysis of a multicenter randomized controlled trial

**DOI:** 10.1186/1471-2369-13-99

**Published:** 2012-09-03

**Authors:** Michael Osthoff, Vanja Piezzi, Theresia Klima, Andreas Christ, Ivana Marana, Sabine Hartwiger, Tobias Breidthardt, Giancarlo Marenzi, Marten Trendelenburg, Christian Mueller

**Affiliations:** 1 Laboratory of Clinical Immunology, Department of Biomedicine, University Hospital Basel, Basel, Switzerland; 2 Clinic for Internal Medicine, University Hospital Basel, Basel, Switzerland; 3 Department of Nephrology, University Hospital Basel, Basel, Switzerland; 4 Department of Anesthesiology, Kantonsspital Olten, Switzerland; 5 Centro Cardiologico Monzino, Milan University, Milano, Italy

**Keywords:** Complement, Mannose-binding lectin, Contrast-induced nephropathy, Ischemia/reperfusion injury, Acute kidney injury

## Abstract

**Background:**

Local renal ischemia is regarded as an important factor in the development of contrast-induced nephropathy (CIN). Mannose-binding lectin (MBL) is involved in the tissue damage during experimental ischemia/reperfusion injury of the kidneys. The aim of the present study was to investigate the association of MBL deficiency with radiocontrast-induced renal dysfunction in a large prospective cohort.

**Methods:**

246 patients with advanced non–dialysis-dependent renal dysfunction who underwent radiographic contrast procedures were included in the study. Baseline serum MBL levels were analyzed according to the occurrence of a creatinine-based (increase of ≥0.5 mg/dL or ≥25% within 48 hours) or cystatin C-based (increase of ≥10% within 24 hours) CIN.

**Results:**

The incidence of creatinine-based and cystatin C-based CIN was 6.5% and 24%, respectively. MBL levels were not associated with the occurrence of creatinine-based CIN. However, patients that experienced a cystatin C increase of ≥10% showed significantly higher MBL levels than patients with a rise of <10% (median 2885 (IQR 1193–4471) vs. 1997 (IQR 439–3504)ng/mL, p = 0.01). In logistic regression analysis MBL deficiency (MBL levels≤500 ng/ml) was identified as an inverse predictor of a cystatin C increase ≥10% (OR 0.34, 95% CI 0.15-0.8, p = 0.01).

**Conclusion:**

MBL deficiency was associated with a reduced radiocontrast-induced renal dysfunction as reflected by the course of cystatin C. Our findings support a possible role of MBL in the pathogenesis of CIN.

## Background

Contrast-induced nephropathy (CIN) is the third leading cause of acute renal failure in hospitalized patients as a result of the expanded use of iodinated contrast media (CM). Consequently, this iatrogenic complication is associated with prolonged hospitalization, significant morbidity and mortality, and increased health care costs [1,2]. The incidence of CIN is higher in patients with predisposing risk factors such as renal impairment, diabetes mellitus, advanced age, congestive heart failure, simultaneous use of nephrotoxic drugs, hypovolemia or large amounts of CM [3].

The pathophysiology of CIN is complex and not fully understood. Yet, evidence from numerous studies suggests that a combination of several mechanisms is responsible for the development of CIN [4]. Local renal ischemia is a direct result of CM induced prolonged vasoconstriction, which primarily affects the outer medulla. Hypoxic injury to this region is aggravated by an increased tubular cell oxygen demand after administration of CM. Consequently, oxidative stress, which enhances the production of reactive oxygen species (ROS) and triggers a local inflammatory response, may cause additional cell injuries during the reperfusion phase [5]. The second important effect of CM involves direct cytotoxic damage to renal tubular cells.

Mannose-binding lectin (MBL) is an innate pattern-recognition, multimeric protein of the complement system that is primarily synthesized in the liver. Several mutations in the *MBL2* gene negatively influence the concentration of circulating functional MBL multimers. Hence, MBL deficiency can be observed in up to 30% of the Caucasian population [6]. Binding of MBL to certain sugar residues on pathogens leads to the activation of the lectin pathway of the complement cascade and subsequently to killing or phagocytosis of microorganisms. Furthermore, MBL is also involved in the binding and removal of dying cells, e.g. after ischemic injury [7,8].

With regard to ischemia and oxidative stress several studies have highlighted the crucial role of MBL in aggravating the inflammatory response and tissue damage during ischemia/reperfusion (I/R) injury of the heart [9-11], the brain [12,13], as well as the kidney [14-16]. However, comparable data in human CIN are scarce. In a recent study Wang et al. have shown that MBL was strongly upregulated in the urine proteome profiles after application of CM [17]. Moreover, in patients with CIN urine levels of MBL significantly increased after the procedure whereas MBL levels remained stable in patients who did not develop CIN. These results suggest that the lectin pathway might be involved in the pathogenesis of human contrast-medium-induced kidney injury. Hence, the current study tested the hypothesis that MBL deficiency is associated with a reduced incidence of CIN.

## Methods

### Study design

Post-hoc analysis of a multicenter, randomized, open-label, controlled trial (Clinicaltrials.gov Identifier: NCT00130598) that compared three different prevention procedures of CIN between March 2005 and December 2009 [18]. The primary endpoint of the study was the maximum change in estimated glomerular filtration rate (eGFR) within 48 hours.

### Study population

In the original trial 273 admitted patients with renal dysfunction, who were scheduled to receive radiographic CM during diagnostic or therapeutic procedures within the next 24 hours, were randomized to three prevention regimens. Subsequently, 258 patients were included in the final analysis. The study was conducted according to the principles of the revised Declaration of Helsinki, had been approved by the local ethical committees (Ethikkommission beider Basel, Basel/Liestal, Switzerland; Comitato Etico Centro Cardiologico Monzino, Milano, Italy), and all participants gave written informed consent for the study. The aim of the original trial was to investigate two regimens of sodium bicarbonate vs. standard volume supplementation with isotonic sodium chloride in the prevention of CIN.

Renal dysfunction was defined as serum creatinine level >93 μmol/L for women and >117 μmol/L for men or eGFR <60 mL/min/1.73 m^2^ as assessed 24 hours before the radiographic procedure. Patients were excluded if they were <18 years old, pregnant, or allergic to radiographic contrast, were undergoing dialysis, had severe heart failure (NYHA III-IV), had taken N-acetylcysteine ≤24 hours before CM administration, or had a clinical vulnerable condition requiring continuous fluid therapy (e.g. severe sepsis).

Patients were randomly assigned to one of three preventive regimens which are outlined in more detail in the original study [18] (Group A: Standard 24 h sodium chloride; group B: 7 h sodium bicarbonate; group C: Short-term sodium bicarbonate).

### Definition of endpoints

The primary endpoint for this study was the development of CIN. Multiple definitions have been used to quantify renal damage after CM. Creatinine-based CIN was defined as an increase of ≥44 μmol/L or ≥25% in serum creatinine concentration within 48 hours after exposure to CM. Cystatin C is an evolving marker for the prediction and diagnosis of CIN. Several studies suggest that serum cystatin C is a more sensitive and rapid indicator of changes in GFR than serum creatinine [19]. In a recent study, a cystatin C increase of ≥10% 24 hours after exposure to CM was found to reliably predict the occurrence of CIN and future adverse events (death from any cause and chronic dialysis) [20]. Therefore, we included this cut-off for the definition of cystatin-based CIN in our secondary analysis. Serial serum cystatin C samples were available from 241 of 258 patients. Furthermore, time to hospital discharge, in-hospital morbidity and mortality, renal replacement therapy and mortality at 90 days were included as secondary endpoints.

### Assessment of renal function

Peripheral venous blood samples for the measurement of creatinine and cystatin C were obtained on the day preceding the scheduled administration of CM and in the morning of the subsequent two days after the contrast exposure. The measurement of these parameters is outlined in more detail in the original study [18].

The eGFR was calculated by means of the abbreviate *Modification of Diet in Renal Disease* (MDRD) equation [21].

### Determination of mannose-binding lectin levels

For the MBL level analysis baseline serum samples were available from 246 of 258 patients. MBL serum concentrations were measured on the same day blinded to any patient data using a commercially available Sandwich-ELISA Kit (*MBL Oligomer ELISA KIT 029*, Lucerna Chem, Luzern, Switzerland) as described previously [22].

Depending on the clinical setting several cut-off values for MBL deficiency have been proposed ranging from 100 to 1000 ng/mL [6]. Thus, we first analyzed the predefined endpoints in relation to MBL levels, and only in a secondary analysis chose a threshold of ≤500 ng/mL for the definition of MBL deficiency, as this cut-off has been shown to reliably predict low-producing *MBL2* genotypes [23].

### Statistical analysis

Continuous data are expressed as means (standard deviation) or median (interquartile range (IQR)) where appropriate, whereas categorical variables are summarized as frequency (percentage). Differences in patient characteristics and outcome measures according to MBL serostatus were analyzed using the Fisher`s exact test or the Mann–Whitney-*U*-Test where appropriate. Due to the non-*Gaussian* distribution of human MBL levels (as determined by Q-Q plot and normality tests) two-group comparison of serum MBL levels were performed using a Mann–Whitney-*U*-test, whereas a Kruskal-Wallis one-way analysis of variance or Friedman test was used for multigroup comparison where appropriate. Dunn’s post test was used to correct *p*-values for multiple comparisons.

Stepwise logistic regression models were used to estimate the effect of MBL levels on predefined endpoints in multivariate analyses after adjustment for covariables with univariate P values less than 0.1. Covariables tested in univariate analysis included patient age, sex, baseline renal function (as assessed by serum creatinine or cystatin C), history of vascular risk factors, acute myocardial infarction or congestive heart failure, amount of administered CM, medication use, prevention regimen (B or C versus A) and baseline blood pressure. All testing was two-tailed, and *p* values less than 0.05 were considered to be statistically significant. All statistical analyses were performed with the use of SPSS for Windows, version 15.0 (SPSS), and Prism for Windows, version 4 (GraphPad).

## Results

### Patients’ characteristics and mannose-binding lectin serum levels

The analyzed study cohort consisted of 246 elderly patients (mean age 75 years) with renal dysfunction who underwent intravenous or intraarterial contrast procedures. Median eGFR at baseline was 44 (IQR 35–52) mL/min/1.73 m^2^. The majority of patients underwent either computer tomography (45%) or cardiac catheterization with or without PCI (21 and 24%, respectively), and 100 (IQR 80–160) mL of CM was administered on average during contrast procedures. The median MBL serum level was 2150 (IQR 560–3794) ng/mL, and 57 of 246 (23%) individuals showed levels ≤500 ng/mL. Similar MBL concentrations were observed in the three intervention groups and in patients with a baseline eGFR of <30, 30–59, and ≥60 ml/min/1.73 m^2^ (data not shown). Important clinical characteristics of the whole study cohort and classified according to the chosen threshold for the definition of MBL deficiency are summarized in Table 1. With the exception of pre-procedural diastolic blood pressure and use of non-steroidal anti-inflammatory drugs patients with MBL levels ≤500 ng/mL did not differ significantly from patients with MBL levels >500 ng/mL with regard to baseline characteristics.

**Table 1:** Clinical characteristics of patients

**Baseline characteristics**	**All**	**MBL ≤500 ng/mL**	**MBL >500 ng/mL**	**P-value**
	(n = 246)	(n = 57)	(n = 189)	
Age in years, mean (SD)	75 (9.4)	74 (10.5)	75 (9.5)	0.4
Female sex, n (%)	89 (36)	24 (42)	65 (34)	0.4
MBL in ng/mL, median (IQR)	2150 (560–3794)	127 (11–330)	2855 (1701–4347)	n/a
BMI in kg/m^2^, median (IQR)	26 (23–29)	27 (24–30)	26 (23–29)	0.09
Contrast medium in mL, median (IQR)	100 (80–160)	100 (80–150)	100 (80–170)	0.3
Medical history				
Hypertension, n (%)	204 (83)	48 (84)	156 (83)	0.8
D.m., n (%)	90 (37)	19 (33)	71 (38)	0.6
Dyslipidemia, n (%)	145 (59)	34 (60)	111 (59)	1.00
Coronary heart disease, n (%)	143 (58)	31 (54)	112 (59)	0.5
CHF, n (%)	111 (45)	23 (40)	88 (47)	0.5
Peripheral artery disease, n (%)	64 (26)	11(19)	53 (28)	0.2
Current smoking, n (%)	40 (16)	7 (12)	33 (17)	0.4
Medication				
ACE-Inhibitor, n (%)	108 (44)	23 (40)	85 (45)	0.7
AT-II-Inhibitor, n (%)	69 (28)	13 (23)	56 (30)	0.40
Diuretics, n (%)	173 (70)	41 (72)	132 (70)	0.9
NSAIDs, n (%)	8 (3)	5 (9)	3 (2)	**0.02**
Baseline parameters				
Systolic BP in mmHg, median (IQR)	130 (120–146)	132 (120–149)	130 (117–145)	0.1
Diastolic BP in mmHg, median (IQR)	70 (60–80)	76 (65–80)	70 (60–80)	**0.01**
Creatinine in μmol/L, median (IQR)	137 (114–162)	140 (113–154)	136 (114–164)	0.9
Cystatin C in mg/L, median (IQR)	1.7 (1.5-2.0)	1.8 (1.5-2.0)	1.7 (1.5-2.1)	0.8
eGFR in mL/min/1.73 m^2^, median (IQR)	44 (35–52)	44 (35–51)	44 (35–53)	0.8
Type of procedure				
Coronary angiogram, n (%)	110 (45)	23 (40)	87 (46)	0.5
PCI, n (%)	52 (21)	12 (21)	40 (21)	1.00
CT scan, n (%)	110 (45)	29 (51)	81 (43)	0.3
Other, n (%)	26 (11)	5 (9)	21 (11)	0.8
Intervention group				
Standard 24 h sodium chloride, n (%)	85 (35)	16 (28)	69 (37)	0.3
7 h sodium bicarbonate, n (%)	82 (33)	22 (39)	60 (32)	0.3
Short-term sodium bicarbonate, n (%)	79 (32)	19 (33)	60 (32)	0.9

P-values are given for the difference between MBL ≤500 and >500 ng/ml. Abbreviations: D.m. = Diabetes mellitus; IQR = interquartile range; BMI = body mass index; CHF = congestive heart failure class I/II by New York Heart Association classification; NSAIDs = non-steroidal anti-inflammatory drugs; eGFR = estimated glomerular filtration rate; CT scan = computer tomography scan. PCI = percutaneous coronary intervention; BP = blood pressure; SD = standard deviation.

### Mannose-binding lectin and creatinin-based contrast-induced nephropathy

**Figure 1:**
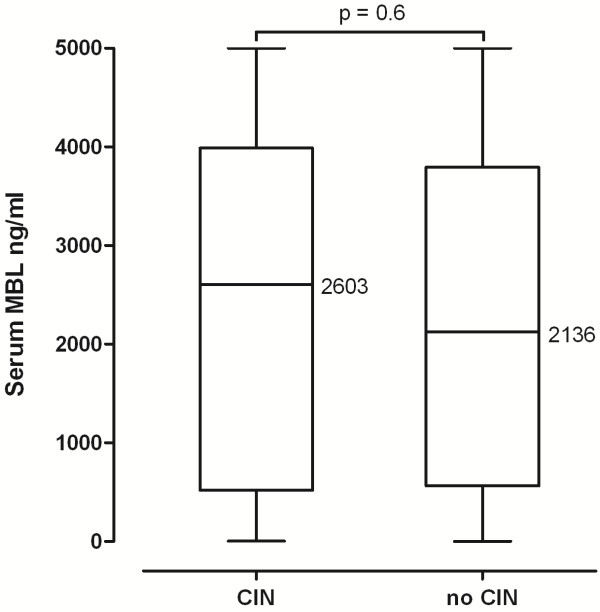
** Differences in MBL serum concentrations according to the occurrence of creatinine-based CIN (defined as increase in serum creatinine concentration of ≥44 μmol/L or 25% within 48h).** Horizontal lines represent medians.

The incidence of CIN, defined as a maximum increase in serum creatinine concentration of more than 44 μmol/L or 25% within 48 hours after exposure to CM, was 6.5% (16/246) in the whole study cohort. In individuals with CIN median serum creatinine levels rose from 140 μmol/L (IQR 103–164) at baseline to 170 μmol/L (110–202) and 202 μmol/L (125–266) at 24 and 48 hours, respectively (p = 0.02 for baseline vs. 48 hours), whereas cystatin C levels had already increased significantly from 2.06 mg/L (IQR 1.38-2.48) at baseline to 2.65 mg/L (1.77-3.10) at 24 hours (p = 0.038). MBL levels in patients who experienced an acute deterioration of renal function did not differ significantly from patients that showed stable creatinine concentrations (median 2603 (IQR 519–3988) vs. 2126 (563–3794) ng/mL, p = 0.6, Figure 1). Results were similar when each intervention group was analyzed separately (data not shown).

With respect to the predefined MBL cut-off 3/57 (5.3%) patients with MBL deficiency developed CIN within 48 hours as compared to 13/189 (6.9%) patients with MBL levels >500 ng/mL (p = 1.0).

### Mannose-binding lectin and cystatin C-based contrast-induced nephropathy

**Figure 2:**
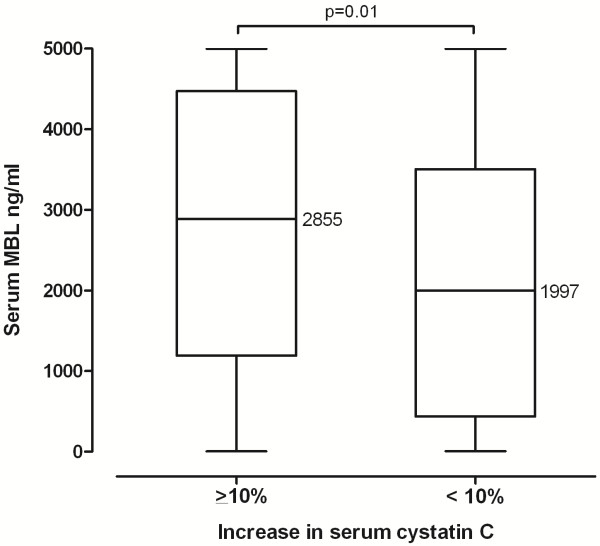
** Differences in MBL serum concentrations according to the occurrence of a serum cystatin C increase ≥10% after exposure to contrast media.** Horizontal lines represent medians.

Recently, an increase of cystatin C ≥10% after exposure to CM was identified as an independent marker for the development of CIN or future adverse events [20]. In our study population serum cystatin C levels rose ≥10% in 58/241 (24%) individuals after undergoing contrast procedures. In these subjects median serum creatinine levels did not change significantly from baseline (132 μmol/L (IQR 112–150)) to 24 hours (133 μmol/L (IQR 111–170)) and 48 hours after exposure to CM (141 μmol/L (IQR 113–187); p = 0.54), and 13 of these 58 individuals simultaneously met the definition of creatinine-based CIN as mentioned above. Serum MBL levels were significantly higher in patients with an increase of serum cystatin C ≥10% compared to patients with a rise of <10% (median 2885 (IQR 1193–4471) vs. 1997 (IQR 439–3504) ng/mL, p = 0.01) (Figure 2). This association could be observed to a similar extent in each of the three intervention groups reaching statistical significance in group A and C (data not shown). Furthermore, 50/185 (27%) MBL sufficient patients, i.e. >500 ng/mL, developed a cystatin C increase of ≥10% after the contrast procedure compared to only 8/56 (14%) patients with MBL deficiency (p = 0.05). 

After adjusting for potential confounders MBL deficient phenotype (i.e. <500 ng/mL) remained associated with a reduced risk of a cystatin C increase of ≥10% (OR 0.34 (95% CI 0.15-0.80), p = 0.01, Table 2). In addition, higher MBL levels were identified as risk factor for a cystatin C increase >10% when MBL levels were entered as continuous variable into the analysis (OR 1.32 (95% CI 1.10-1.58) for every 1000 ng/mL increase, p = 0.003).

**Table 2:** Independent predictors of a cystatin C increase >10% after exposure to CM

**Variable**	**OR**	**95% CI**	**P-value**
MBL deficient phenotype (<500 ng/ml)	0.34	0.15-0.80	0.01
Prevention regimen			
Standard 24 h sodium chloride	1		
7 h sodium bicarbonate	3.40	1.52-7.60	0.003
Short-term sodium bicarbonate	2.93	1.28-6.70	0.01
Baseline diastolic blood pressure^1^	1.03	1.00-1.05	0.054

Variables entered into the stepwise logistic regression analysis were age, sex, baseline cystatin C, history of vascular risk factors, acute myocardial infarction, congestive heart failure, amount of administered contrast medium, medication use, prevention regimen and baseline blood pressure.

^1^ per mmHg.

### Mannose-binding lectin and clinical outcomes

Follow-up was completed in all patients included in this analysis. In-hospital morbidity and mortality, dialysis dependency and mortality at 3 months did not differ significantly between MBL deficient and sufficient individuals (Table 3). There was a non significant trend towards a longer hospital stay in MBL sufficient individuals. In addition, median MBL levels were significantly higher in patients who were readmitted for cardiac reasons within 3 months after administration of CM (median 3059 (IQR 1392–4218) vs. 1899 (IQR 467–3727), p = 0.03), whereas there was no difference in MBL levels with regard to in-hospital morbidity and mortality, dialysis dependency and mortality at 3 months (data not shown).

**Table 3:** Clinical outcomes dependent on MBL serotype

**Clinical Outcome**	**MBL ≤500 ng/ml (n = 57)**	**MBL >500 ng/ml (n = 189)**	**P value**
Length of stay in days, median (IQR)	6 (3–13)	8 (3–16)	0.07
In-hospital morbidity and mortality, n (%)	4 (7)	6 (3)	0.25
Dialysis dependency at 3 months, n (%)	2 (4)	2 (1)	0.23
Hospitalization for cardiac cause at 3 months, n (%)	5 (9)	34 (18)	0.10
Mortality at 3 months, n (%)	8 (14)	18 (10)	0.33

## Discussion

The lectin pathway of the complement system has been shown to considerably contribute to tissue damage during ischemia/reperfusion injury of several organs, including the kidneys [14,16]. This is the first study to assess the importance of serum MBL with respect to the development of CIN in a clinical trial. MBL deficiency did not influence the occurrence of CIN as defined by a commonly quoted serum creatinine increment. However, it was associated with a limited increase in Cystatin C after the administration of CM.

To our knowledge the role of MBL has not been investigated in a rodent model of CIN. However, the fact that local ischemia and reperfusion is at least in part responsible for the development of CIN [4], and that MBL aggravates tissue damage during I/R injury [9,11,12,15], suggests a role of MBL in the pathogenesis of contrast-induced acute kidney injury.

With regard to the primary endpoint, the incidence of creatinine-based CIN, several reasons might account for the absent effect of MBL deficiency in this study. According to the baseline characteristics, the study cohort included only a rather small percentage of very high-risk patients (as reflected by 29/246 (11.7%) with GFR <30 mL/min/1.73 m^2^, 52/246 (21.1%) with coronary angiography and ad hoc PCI, 69/246 (28.0%) with contrast media volume ≥140 mL [24], 90/246 (36.6%) with diabetes mellitus, and the exclusion of patients with advanced congestive heart failure), and CIN was diagnosed infrequently, especially in the sodium chloride prevention group (incidence 1.2%). Due to the latter fact the analysis of smaller differences between MBL sufficient and deficient patients is limited. Furthermore, in this study cohort low to moderate CM-induced damage is probably not reflected by a decline in kidney function as measured by the serum creatinine concentration, a parameter with limited sensitivity to detect an acute deterioration in renal function within 48 hours [25]. Besides underestimating the true change in GFR, the increase in serum creatinine after CM exposure is delayed achieving a maximum two to five days after CM exposure as compared to cystatin C, a more sensitive marker, which was shown to rise earlier, to peak as early as 24 hours after CM administration, and to detect even subtle changes in GFR after acute kidney injury including CIN [19,26-30]. Indeed, when we analyzed the influence of MBL deficiency on the cystatin C course after CM exposure we observed a remarkable association: As compared to patients with MBL levels >500 ng/mL subjects with MBL deficiency were almost two-times less likely to develop a cystatin C increase ≥10% after administration of CM, a cut-off that has recently been proposed as an independent diagnostic and prognostic tool with respect to the occurrence of CIN and future adverse events [20]. This suggests that deficiency of MBL might attenuate some of the detrimental effects of CM. Though being small in magnitude this increase in cystatin C of ≥10% might have important consequences for the patients as even apparently minor decreases in renal function have been shown to be associated with excessive mortality rates independent of other known risk factors [20,31]. However, we were not able to demonstrate a consistent association of MBL deficiency with superior clinical outcomes, which the study was not powered for.

On the other hand, the importance of renal I/R injury in pathogenesis of CIN might be overestimated as several other factors (including direct toxic effect on renal tubular cells, increased urinary viscosity, and tubular obstruction) influence kidney function after exposure to CM [32]. Moreover, I/R models that demonstrated a pivotal role of MBL in the context of renal ischemia (i.e. transient or permanent occlusion of a major renal artery) are most likely not comparable to the complex events occurring after administration of CM. The duration and extent of ischemia might be crucial as well. In a rodent model of renal I/R activation of the MBL-pathway was not induced by warm ischemia of less than 30 minutes [14]. Data on the duration and significance of renal vasoconstriction and hypoxia after CM exposure in humans remain controversial [4]. However, it has been demonstrated that MBL and its associated protease mannose-binding protein-associated serine protease 2 are strongly upregulated in the post-procedural urinary proteome profiles after application of CM, and that urine levels of MBL significantly increase in patients who developed CIN whereas MBL levels remain stable in non-CIN patients [17]. Finally, the alternative pathway has been implicated in experimental renal ischemia/reperfusion injury and might play a dominant role in humans as compared to the MBL pathway [33].

Despite the randomized controlled trial design of the original study and the precisely characterized cohort the present study has important limitations including the post hoc analysis of MBL serum levels and the use of a surrogate marker as primary endpoint. Furthermore, limiting the measurement of serum creatinine to 48 hours after CM exposure might result in an underestimation of creatinine-based CIN. As genetic material was not available, analysis of MBL deficiency solely relied on MBL phenotype. However, MBL serum levels show little variation throughout life, and correlate well with the functional activity of the MBL pathway *in vivo*[34]. As individuals with the same genotype may vary up to tenfold in MBL serum levels, measurement of MBL serum levels by ELISA might in fact represent a more reliable approach than determination of genotypes [35,36]. MBL levels (as measured by sandwich ELISA) have been shown to be significantly increased in Asian pre-dialysis and dialysis patients compared to healthy controls [37,38]. To the best of our knowledge comparable studies in a Caucasian population with moderate renal impairment (similar to our study patients) are lacking.

## Conclusion

MBL deficiency did not influence the development of CIN in this study cohort as defined by the course of serum creatinine. However, it was associated with a reduced radiocontrast-induced renal dysfunction as assessed by cystatin C, a more sensitive and reliable parameter for estimating GFR early after CM exposure. Additional studies in patients at high risk for CIN are needed to fully elucidate the role of MBL in the pathogenesis of human CIN.

## Competing interests

The authors declare that they have no competing interests.

## Authors’ contributions

MO, VP, MT, and CM were the principal investigators and take primary responsibility for the paper. CM and MT designed the study. TK, AC, IM, SH, and TB recruited the patients and collected data. MO and VP performed the laboratory work for this study, analyzed and interpreted data, and wrote the article. TK, AC, IM, SH, TB, GM, MT, and CM provided intellectual content, revised the article, and gave final approval.

## Pre-publication history

The pre-publication history for this paper can be accessed here:

http://www.biomedcentral.com/1471-2369/13/99/prepub
